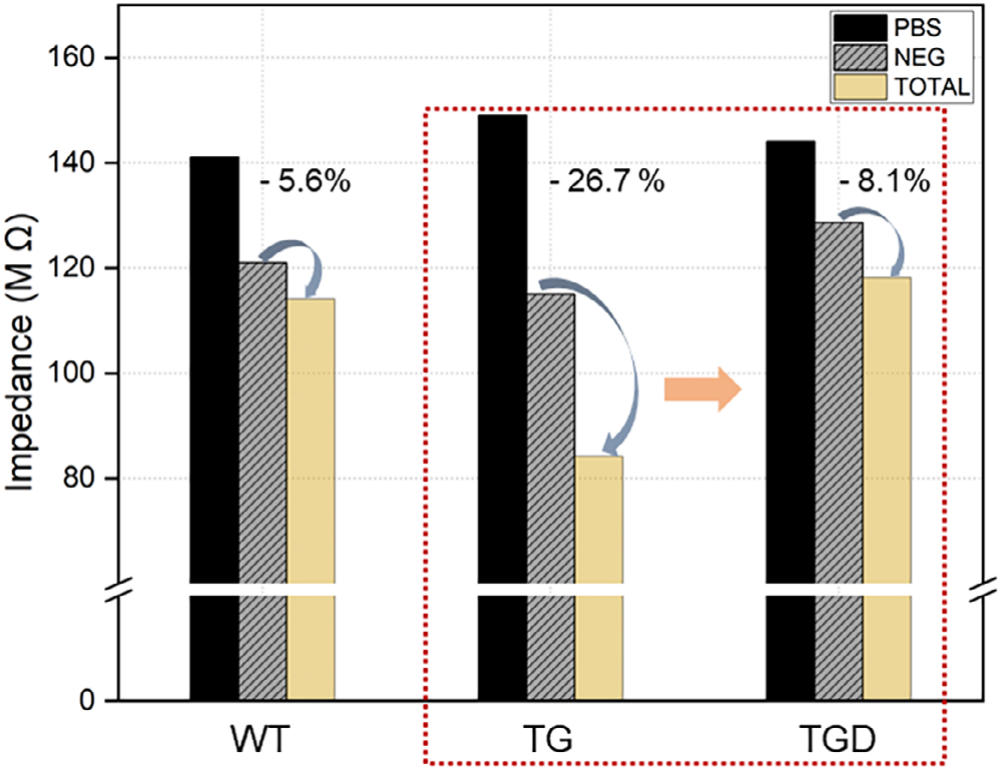# Measurement of Blood GFAP Concentration to Evaluate KDS2010 (MAO‐B Inhibitor) Drug in APP/PS1 Mice Using a Magnetic Bead‐based Electrochemical Sensor

**DOI:** 10.1002/alz.092221

**Published:** 2025-01-09

**Authors:** Hana Cho, Sang‐Heon Park, Yeeun Jeong, Jong‐Hyun Park, Soo Hyun Lee

**Affiliations:** ^1^ Korea Institute of Science and Technology, Seoul Korea, Republic of (South); ^2^ KIST school, University of Science and Technology, Seoul Korea, Republic of (South)

## Abstract

**Background:**

Blood GFAP levels have the potential to reflect and predict worsening disability in individuals with degenerative diseases such as Alzheimer’s Disease (AD) and Parkinson’s Disease (PD). Recently published research suggests that blood GFAP levels can be used to detect even subtle damage to the degenerative disease. In this study, we evaluated the effectiveness of the KDS2010 (MAO‐B inhibitor) drug targeting AD by measuring blood glial fibrillary acidic protein (GFAP) levels in APP/PS1 mice using a magnetic bead‐based electrochemical sensor.

**Method:**

Magnetic beads were coated with a primary antibody that was specifically bound to the antigen. Subsequently, the magnetic beads were incubated with 3 blood different samples from: 1) wild‐type (control, WT), 2) APP/PS1 model (TG), and 3) APP/PS1 + KDS2010 drug (TGD). The magnetic beads coated with the samples were measured using a nano‐gap based electrochemical sensor.

**Result:**

The TG sample exhibited much lower impedance values compared to the WT sample due to the high concentration of GFAP in the blood. On the other hand, the TGD samples showed a value (‐8.1%) similar to that (‐5.6%) of the WT samples, as shown in Figure 1. This result means that KDS 2010 (MAO‐B inhibitor) plays a good role in suppressing the reactivity of astrocytes. And then, blood GFAP levels can be lower after KDS 2010 treatment.

**Conclusion:**

We validated the effectiveness of the KDS2010 in APP/PS1 mice with a magnetic bead‐based electrochemical sensor. Successful validation of a point‐of‐care platform for blood GFAP could increase the diagnostic reliability of AD, which could have a significant economic impact on the dementia diagnostics market.